# Multiple Eruptive Dermatofibromas: A Case Series and Review of Diagnostic Challenges and Systemic Associations

**DOI:** 10.7759/cureus.77929

**Published:** 2025-01-24

**Authors:** Kotaiba Alenezy, Yousef Dashti, Maryam Dashti, Manar Alenezi

**Affiliations:** 1 Dermatology, Mubarak Al-Kabeer Hospital, Jabriya, KWT

**Keywords:** autoimmune diseases, clinical dermatology, dermatofibroma, dermatofibroma variants, dermoscopy, histopathology, immunohistochemistry, multiple eruptive dermatofibromas, rare skin conditions, systemic associations

## Abstract

Multiple eruptive dermatofibromas (MEDF) is a rare dermatological condition characterized by the sudden appearance of multiple benign skin lesions, often associated with systemic or autoimmune conditions. This case series describes three female patients with MEDF, highlighting their distinct clinical features and systemic associations. Diagnoses were confirmed through consistent histopathological findings, demonstrating the importance of integrating clinical, dermoscopic, and histopathological evaluations in diagnosing MEDF. The differential diagnosis included other dermatological conditions with overlapping features, which were excluded based on clinical and pathological findings. This report emphasizes the potential role of immune dysregulation and genetic predisposition in MEDF pathogenesis. It underscores the need for further research to understand its mechanisms better, improve diagnostic accuracy, and establish standardized management approaches.

## Introduction

Dermatofibromas are common benign skin growths. They typically present as hyperpigmented papules or nodules on the legs and occur most often in the third and fourth decades of life. Multiple eruptive dermatofibromas (MEDF) is a rare dermatological phenomenon characterized by the development of multiple dermatofibromas within a short period of time [1]. While typical dermatofibromas are solitary, benign lesions, MEDF poses unique diagnostic challenges due to its unusual clinical presentation and potential overlap with other cutaneous or systemic conditions. Although the etiology of MEDF remains unclear, it has been reported in association with autoimmune conditions, immunosuppressive therapies, and, rarely, genetic predispositions. To our knowledge, fewer than 100 cases of MEDF have been reported in the literature since its initial description in 1970.

This case series describes three female patients with MEDF, highlighting distinctive clinical and systemic findings, including autoimmune markers and comorbidities. The objective is to emphasize the diagnostic complexity and systemic associations of MEDF and to contribute to the limited literature on this rare condition.

## Case presentation

Case 1

**Figure 1:**
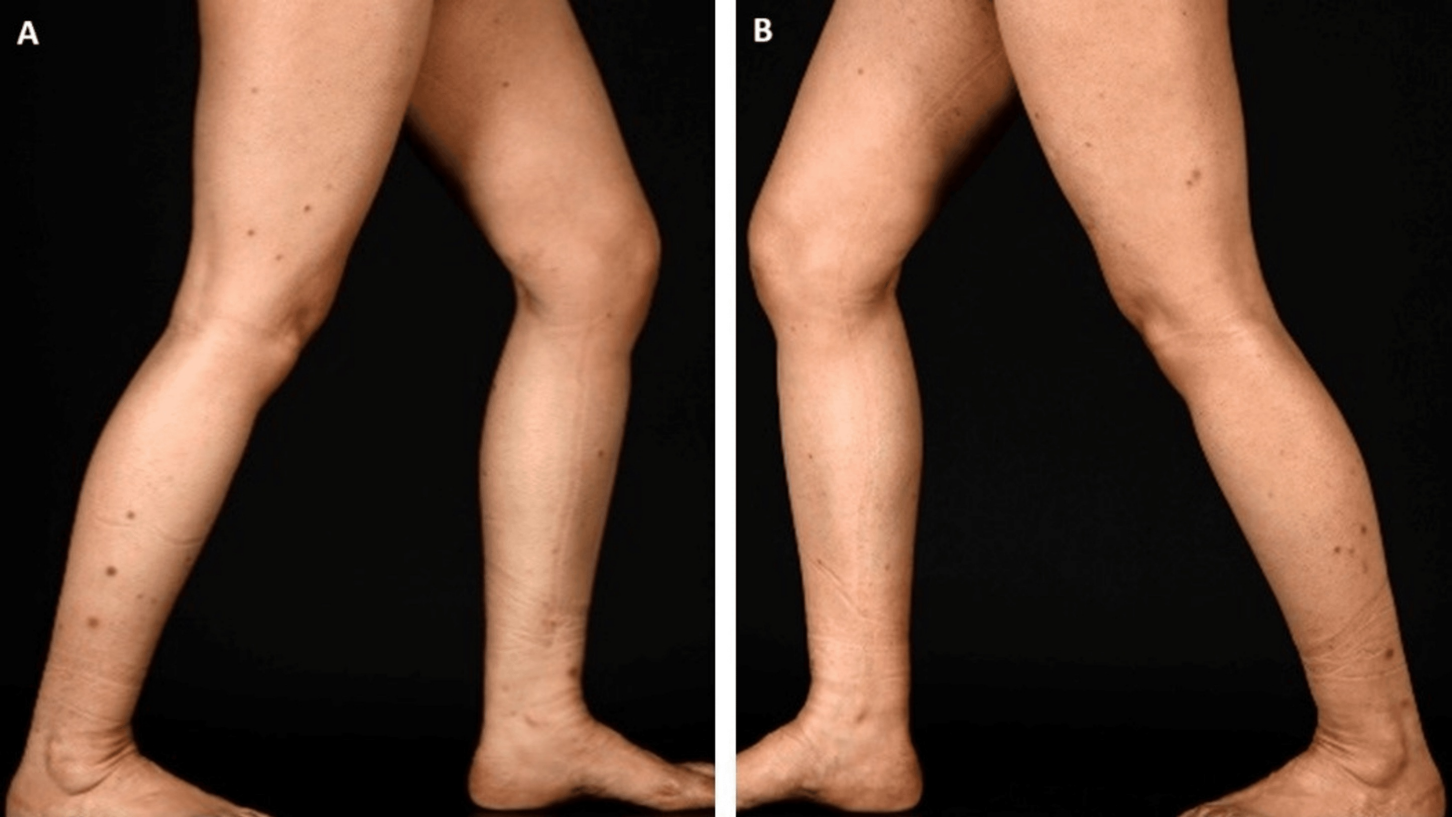
Case 1 clinical view of the (A) right side and (B) left side of the legs showing pigmented papular lesions

A 39-year-old female with a medical history notable for sickle cell trait, hay fever, and tuberculosis presented with progressive onset of darkly pigmented papular lesions on her lower extremities over the past few years. Physical examination revealed multiple firm pigmented papules and nodules, predominantly on the legs' anterolateral aspect (Figure 1A-1B). The dermoscopic evaluation showed a central white scar-like patch surrounded by a peripheral pigment network. Laboratory investigations revealed weakly positive antinuclear antibodies (ANA), anti-Ro antibodies, and a strongly positive Lupus anticoagulant.

Case 2

**Figure 2:**
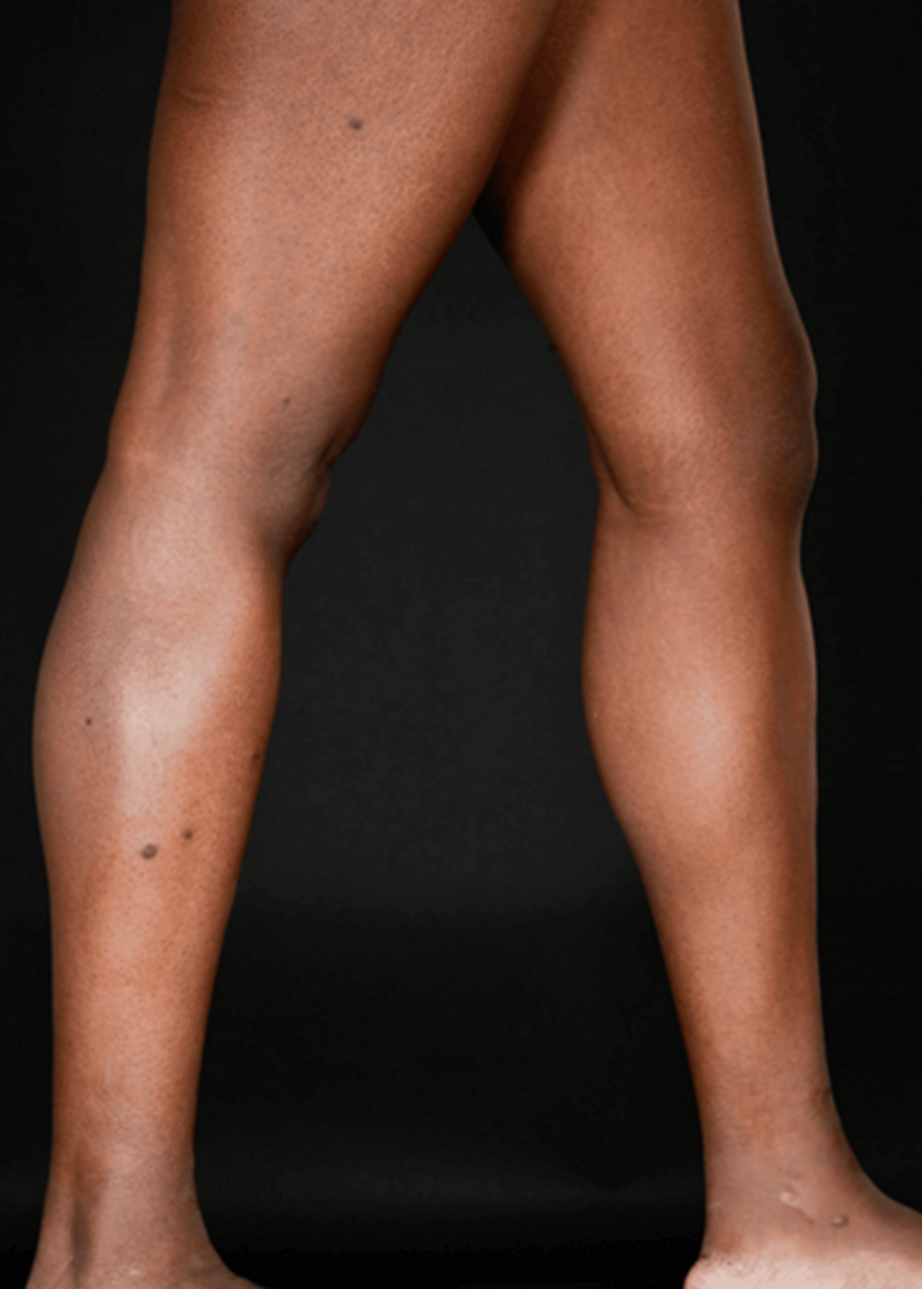
Case 2 clinical view of the right side of the legs showing pigmented papular lesions

A 38-year-old female with a history of Takayasu's arteritis on prednisolone and azathioprine, pulmonary hypertension, hepatic hemangioma, and osteopenia presented with a similar complaint of multiple dark spots, mainly on the right side of her body. Examination revealed hyperpigmented papulo-nodular lesions with a smooth surface and firm consistency, all less than 1 cm in size (Figure 2). Dermoscopic findings were comparable to those observed in Case 1. Laboratory investigations yielded unremarkable results.

Case 3

**Figure 3:**
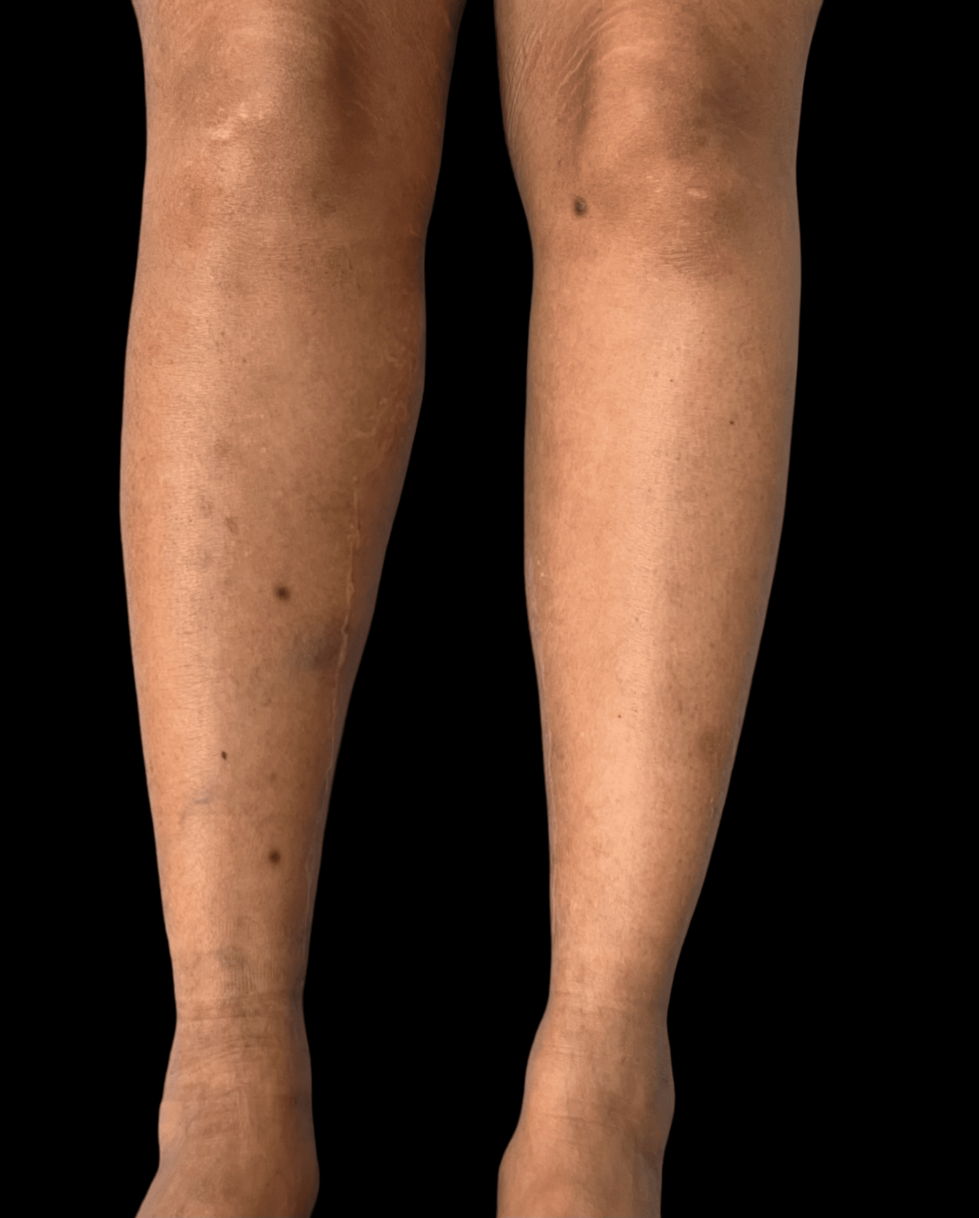
Case 3 clinical view of both legs showing pigmented papular lesions and the right-sided vein harvesting scar

**Figure 4:**
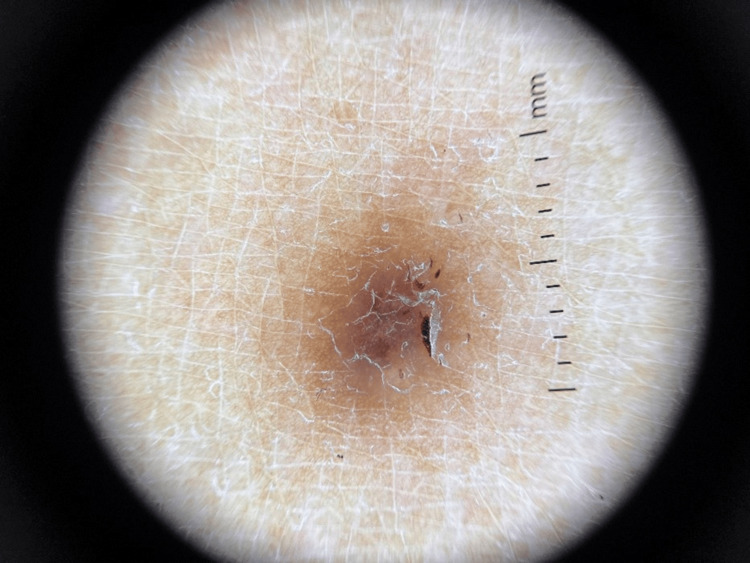
Case 3 dermoscopic image showing typical dermatofibroma features, including a central white scar-like area and peripheral pigment network

A 42-year-old female with a history of fever of unknown origin and arthralgia at age 18, early-onset hypertension, and coronary artery disease requiring heart surgery at age 28. Additionally, she gave birth to a child with Trisomy 22 (cat eye syndrome) at age 32. She exhibited similar lesions, initially on the posterior trunk, which progressively increased in number and size over months. The most recent lesions appeared on both thighs (Figure 3). Dermoscopic examination revealed darkly pigmented papular lesions with a central scar-like patch surrounded by a fine pigment network (Figure 4), consistent across all cases.

**Figure 5:**
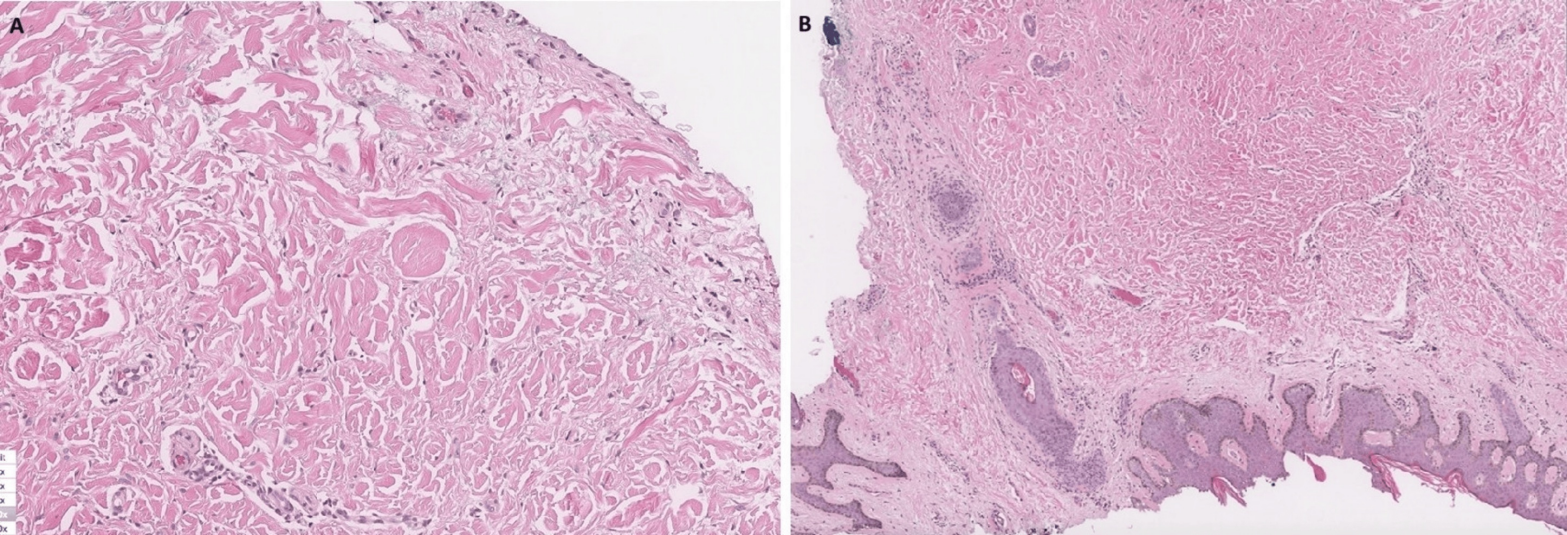
Case 3 H&E histopathology slide of the lesion showing characteristic dermatofibroma features: (A) higher and (B) lower magnification H&E: hematoxylin and eosin

In all three patients, punch biopsy confirmed atrophic fibrous histiocytomas histologically, characterized by a paucicellular dermal spindle cell proliferation within a collagenous background. These findings were consistent with the suspected diagnosis of MEDF. Histology slide images were available only for Case 3 (Figure 5A-5B).

## Discussion

Our case series highlights the diagnostic complexities associated with MEDF. MEDF often presents with multiple lesions that change over time, a behavior uncommon in typical dermatofibromas, which usually appear as solitary and stable papules or nodules. The eruption of multiple lesions can mimic other dermatological conditions, making a precise diagnosis challenging without histopathological confirmation [2]. Each of our cases demonstrated unique clinical features, including pigmentation and rapid lesion proliferation, initially suggestive of other dermatological conditions, emphasizing the importance of a comprehensive differential diagnosis, as shown in Table 1 [3-5].

**Table 1:** Differential diagnoses of MEDF MEDF: multiple eruptive dermatofibromas, DFSP: dermatofibrosarcoma protuberans, HIV: human immunodeficiency virus, AIDS: acquired immunodeficiency syndrome

Condition	Clinical features	Differentiating features	Typical locations	Age of onset/demographics
Dermatofibromas	Solitary papules or nodules	Lack of multiple lesions erupting over time	Anywhere on the body	Adults, slight female predominance
Neurofibromatosis type 1	Cutaneous neurofibromas, which may resemble dermatofibromas	Associated with other clinical features such as café-au-lait macules, neurofibromas in other locations, and Lisch nodules in the iris	Typically, on trunk and extremities	Onset in childhood, autosomal dominant inheritance
Cutaneous lymphoma (e.g., lymphomatoid papulosis or primary cutaneous T-cell lymphoma	May present with multiple papules or nodules	Dermoscopic and histopathologic evaluation can help differentiate these from MEDF	Variable, often on trunk and limbs	Middle-aged to elderly, may have history of immunosuppression or autoimmune disease
Generalized eruptive histiocytoma	Small, dome-shaped lesions with umbilication	Dense dermal histiocyte infiltrate on histology	Trunk, extremities	Usually in adults
DFSP	Firm, often painless nodules or plaques	Infiltrative growth pattern, CD34-positive staining	Typically on trunk and extremities	Adults, slight male predominance
Leiomyoma	Smooth, firm papules or nodules	Arising from smooth muscle, presence of fascicles on histology	Usually on trunk and extremities	Adults
Kaposi’s sarcoma	Purple-red macules, papules, or nodules	Associated with human herpesvirus 8lymphatic vessel proliferation	Lower extremities, face, and trunk	Middle-aged to elderly, immunocompromised individuals or those with HIV/AIDS

As observed in our patients, histopathology in MEDF cases often reveals atrophic fibrous histiocytomas with a distinct collagenous background. However, while special immunohistochemical staining was not utilized in our series due to it not being an available modality in our labs at the time, it is important to acknowledge that these techniques can be crucial in differentiating MEDF from other spindle cell neoplasms, such as dermatofibrosarcoma protuberans (DFSP) and leiomyomas [6]. Immunohistochemical markers such as factor XIIIa are typically positive in dermatofibromas, aiding in their identification, while CD34 is characteristically positive in DFSP. This distinction is critical, especially considering DFSP’s potential for local recurrence and its more aggressive behavior. Although clinical presentation and standard histopathology were sufficient to confirm MEDF in our cases, future evaluations could benefit from incorporating immunohistochemical staining when the diagnosis is ambiguous [7].

The association of MEDF with systemic autoimmune conditions presents an intriguing aspect of its pathophysiology. Two of our patients had histories of autoimmune markers or conditions, including Takayasu’s arteritis and positive ANA markers, suggesting an underlying immune dysregulation [4,8]. Previous case studies and literature reviews have noted that MEDF may occur more frequently in patients with autoimmune conditions or those undergoing immunosuppressive therapy, as seen in our case with Takayasu’s arteritis [8]. These findings indicate a potential link between MEDF and immune dysfunction, possibly mediated by chronic inflammatory states that influence dermal fibroblast behavior.

Genetic research has begun to provide insights into MEDF’s potential hereditary basis, with recent studies identifying missense mutations in the F13A1 gene, which encodes factor XIIIa. This mutation is thought to impair fibrin stabilization, potentially leading to abnormal fibroblast proliferation and, consequently, dermatofibroma formation [9]. Anecdotal cases have reported autosomal dominant inheritance patterns among unrelated families with multiple dermatofibromas, further supporting a genetic predisposition. In our series, consistent histopathological findings, including spindle cell proliferation and a collagenous background, align with these reports and warrant further genetic investigation to confirm these mechanisms [10].

## Conclusions

This case series contributes to the limited literature on MEDF, highlighting its diagnostic complexities and potential systemic associations. Given the link between autoimmune markers and potential genetic factors, a multidisciplinary approach involving dermatologists, rheumatologists, genetic counselors, and other relevant healthcare professionals is essential for optimal patient care, particularly in cases with unusual proliferative behaviors. Integrating clinical, dermoscopic, and histopathological evaluations is critical for accurate diagnosis. Further research into the pathophysiology of MEDF, including genetic screening, long-term immunological follow-up, and the development of standardized diagnostic criteria, will be vital to understand better and manage this rare condition.
